# Patient handoffs and multi-specialty trainee perspectives across an institution: informing recommendations for health systems and an expanded conceptual framework for handoffs

**DOI:** 10.1186/s12909-023-04355-5

**Published:** 2023-06-13

**Authors:** Sarah R. Williams, Stefanie S. Sebok-Syer, Holly Caretta-Weyer, Laurence Katznelson, Ann M. Dohn, Yoon Soo Park, Michael A. Gisondi, Ara Tekian

**Affiliations:** 1 Department of Emergency Medicine, Stanford University School of Medicine, 900 Welch Road, Suite 350, Palo Alto, CA 94304 USA; 2 Departments of Neurosurgery and Medicine, Stanford University School of Medicine, Stanford, USA; 3 Graduate Medical Education, Stanford University School of Medicine and Stanford Health Care, Stanford, USA; 4 Department of Medical Education, University of Illinois at Chicago College of Medicine, Chicago, USA

**Keywords:** Handoffs, Transitions of care, Trainee perspectives, Multi-specialty, Blame, Shame

## Abstract

**Background:**

Safe and effective physician-to-physician patient handoffs are integral to patient safety. Unfortunately, poor handoffs continue to be a major cause of medical errors. Developing a better understanding of challenges faced by health care providers is critical to address this continued patient safety threat. This study addresses the gap in the literature exploring broad, cross-specialty trainee perspectives around handoffs and provides a set of trainee-informed recommendations for both training programs and institutions.

**Methods:**

Using a constructivist paradigm, the authors conducted a concurrent/embedded mixed method study to investigate trainees’ experiences with patient handoffs across Stanford University Hospital, a large academic medical center. The authors designed and administered a survey instrument including Likert-style and open-ended questions to solicit information about trainee experiences from multiple specialties. The authors performed a thematic analysis of open-ended responses.

**Results:**

687/1138 (60.4%) of residents and fellows responded to the survey, representing 46 training programs and over 30 specialties. There was wide variability in handoff content and process, most notably code status not being consistently mentioned a third of the time for patients who were not full code. Supervision and feedback about handoffs were inconsistently provided. Trainees identified multiple health-systems level issues that complicated handoffs and suggested solutions to these threats. Our thematic analysis identified five important aspects of handoffs: (1) handoff elements, (2) health-systems-level factors, (3) impact of the handoff, (4) agency (duty), and (5) blame and shame.

**Conclusions:**

Health systems, interpersonal, and intrapersonal issues affect handoff communication. The authors propose an expanded theoretical framework for effective patient handoffs and provide a set of trainee-informed recommendations for training programs and sponsoring institutions. Cultural and health-systems issues must be prioritized and addressed, as an undercurrent of blame and shame permeates the clinical environment.

**Supplementary Information:**

The online version contains supplementary material available at 10.1186/s12909-023-04355-5.

## Background

The transition of patient care between physicians, also commonly referred to as patient handoffs, is a high stakes communication exchange that can pose a significant threat to patient safety. Research suggests that up to 80% of medical errors have been related to miscommunications between providers, particularly during transitions of care (e.g., “sign-outs”) [1]. Poor handoffs frequently cause sentinel events, [2] and numerous healthcare organizations have called for improved handoff processes to ensure patient welfare [3–7]. Challenges included issues around time, setting, the communication process, and education around handoffs [8]. Developing a better understanding of the challenges and barriers faced by those on the front lines of care, our trainees, is critical if we are to authentically address this continued threat to patient safety.

The Joint Commission defines a handoff communication as “a real-time process of passing patient-specific information from one caregiver to another or from one team of caregivers to another for the purpose of ensuring the continuity and safety of the patient’s care” [2 (p. 1)] Unfortunately, studies from multiple specialties have found that handoff practices often deviate from recommended guidelines [9–14]. Mechanisms to ensure safe handoffs are necessary regardless of medical discipline, as duty-hour limitations demand a high frequency of handoffs by trainees [9, 15, 16].

Most handoff literature represents a single specialty’s experiences (such as pediatrics, emergency medicine, internal medicine), or studies of one specialty specifically handing off to another. And while handoff recommendations and protocols are available in the literature, very few are generated from the perspectives of trainees. To our knowledge, none have represented such a wide range of specialties as our study. Therefore, this paper adds to the broader understanding through examination of multi-specialty experiences of handoffs, representing the diverse and rich perspectives of almost 700 trainees representing over 30 specialties concurrently. Through this lens, we can view commonalities around experiences and consider how different specialties are both supported and challenged within the broader system.

Safe and effective physician-to-physician patient handoffs are integral to patient safety. Unfortunately, poor handoffs continue to be a major cause of medical errors. To address this continued patient safety threat, we aim to develop a better understanding of the challenges around handoffs faced by our medical trainees who are on the “front lines” of patient care. Our objectives include: 1) exploring the lived experiences around handoffs for a broad range of trainees representing multiple specialties, 2) providing a set of trainee-informed recommendations (for both training programs and institutions) to improve the process, content, and culture surrounding handoffs, and 3) expanding upon an existing theoretical framework around handoffs that incorporates these additional perspectives to aid in future discussions of this important topic.

### Methods

**Study design.** Using a constructivist paradigm, we conducted a concurrent/embedded design mixed-methods study [17] to investigate trainees’ experiences, attitudes, and opinions of patient handoffs across our large academic medical center. We designed our survey to include both Likert-style selected response questions and open-ended response questions to solicit information about the lived experiences of trainees from multiple specialties. We collected the quantitative and quantitative data concurrently and analyzed the data sequentially starting with the quantitative analysis and then followed up using both inductive and deductive approaches for qualitative data analysis.

**Sensitizing concepts.** We utilized Arora’s Theoretical Framework to Improve Handoffs [18] as our conceptual model, which highlights both “costs of coordination” and “agency problems” [18–21]. Costs of coordination are described as the increased chance of a breakdown in communication between an expanding number of specialists [18–19]. Agency is defined as “physician entrustment to act in the best interest of patients” [18(p. 12), 20–21] Through a case study of a new night float system in an internal medicine residency, Arora mapped these concepts to the Accreditation Council for Graduate Medical Education (ACGME) Core Competencies of Interpersonal Communication Skills (coordination costs) and Professionalism (agency) [6, 18].

**Study context/setting.** The study took place at Stanford University Hospital, a large, suburban, university-affiliated academic medical center and tertiary referral center located in the San Francisco Bay Area of California in the United States. Data was collected June-August 2014, in preparation for our institution’s first Clinical Learning Environment Review (CLER site) visit. At that time, we had 1138 resident and fellow trainees (“housestaff”) and over 95 ACGME-approved training programs. We surveyed these housestaff for their collective handoff experiences. Given our trainees perform the vast majority of patient handoffs at shift/work cycle changes, eligible respondents included all housestaff at our institution. Participation was voluntary, and there were no incentives to participate.

**Data collection.** In order to protect participant anonymity, we collected only two types of demographics: specialty program type and level of training. To further protect anonymity of respondents, we combined years (PGY 4–5, 6–9, > 9) for small programs longer than 3 years duration. We delayed this report of our qualitative dataset such that study respondents transitioned to the next chapter of their careers, further mitigating any potential safety threat.

**Survey instrument development.** We designed the survey instrument to assess our housestaff’s knowledge, skills, attitudes, and experiences regarding several key handoff elements (see Appendix A). Crafting of the survey items was informed by a review of the existing literature using PubMed® as well as trainee assessment tools utilized by our institution’s graduate medical education office.

For this PubMed®literature review, search terms included: “transitions of care”, “transitions of care patient safety”, “sign-out”, “handoff”, “medicine hand off patient safety”, “surgery hand off patient safety”, “transitions of care sign-out hand off emergency medicine”, “duty hours handoffs”, “consults communication”, “consult services communicate”, “electronic medical record physician nurse communication”, “resident perspectives patient handoffs.” These articles were reviewed for relevant content. Bibliographies of the most applicable articles were also reviewed to capture important articles missed in the PubMed® search. The survey was further developed through iterative pilot testing with content experts and stakeholders. To ensure both content and construct validity, stakeholders included residents, faculty, and educators, both through the MHPE Program at UIC and again iteratively with members of the Transitions of Care Task Force at Stanford. This pilot testing was also utilized to identify key content, as well as to streamline survey items, design, and ease-of-use. We explored insights from interdisciplinary faculty, nurses, housestaff, institution quality experts, hospital leadership, and experts in medical education. Survey content was also informed by trainee assessment tools utilized by our institution’s GME office, based on the important work by Leora Horwitz and her team in 2012 [22].

We refined the survey through iterative pilot testing [23] with content experts and other stakeholders such as housestaff, faculty, and educators. These were initially sampled from the Master of Health Professions Education program at the University of Illinois at Chicago, and then again iteratively with members of the Transitions of Care Task Force at Stanford School of Medicine. Pilot testing added to our response process validity, identifying additional key content and streamlining survey items, design, and ease-of-use. We then sought additional feedback from interdisciplinary faculty, nurses, housestaff, institutional quality experts, hospital leadership, and medical education experts [22, 24].

We used our refined instrument to survey trainees about their current handoff practices, barriers to better handoffs, and potential process changes that could improve their current workflows. We asked them to consider the feasibility of strategies given work hour rules and other potential staffing and resource constraints. We queried modifications that could be considered if additional resources were available, such as educational programs, transition of care tools (both verbal and written), and potential utilization of the electronic medical record. We explored trainee attitudes regarding handoff standardization, as well as the utility of certain handoff tools which show promise in this domain, such as the Situation, Background, Assessment, and Recommendation (SBAR) tool [25, 26] and Illness severity, Patient Summary, Action list, Situation awareness and contingency planning, and Synthesis by receiver (I-PASS) tool [12, 27].

An early open-ended question asked respondents to reflect on the specifics of a memorable handoff they received. This question not only explored important content that might have been missed by selected response options, but was also purposely placed early in the survey to increase their level of engagement. It acted as an emotional hook, aiming to increase survey completion [28]. For selected response questions, we used a mix of both forced choice and neutral-response option answer types, depending on the content explored by each question. We reduced social desirability bias by asking respondents to reflect on handoffs they had received or observed, rather than asking them to describe their own behaviors [29]. Other techniques utilized to enhance response rate and reduce nonresponse bias, as described by Phillips, Reddy, and Durning, include providing an electronic, on-line survey, protecting psychological safety, emphasizing the trainees’ authority and expertise in this space, reminders, timing, and salience [30].

**Data analysis.** We used descriptive statistics to report demographic characteristics of respondents and selected response questions. We grouped responses to items such that the responses “very common” and “common” were both considered “consistently performed [a task],” and the responses “highly variable” and “uncommon” were both considered “inconsistently performed [a task].” We analyzed the data using Microsoft Excel (Redmond, WA).

Our study explores a broad, multi-specialty context. Therefore, we initially utilized an inductive approach to ensure potential new themes would be considered. For this, we performed a reflexive thematic analysis of open-ended responses using the 6-step method described by Braun and Clark [31]. The transcripts of the entire qualitative datasets were first reviewed by SRW and SSS to familiarize ourselves with the data. Initial coding of the original free-response survey response datasets was performed line-by-line by two study investigators (SRW, SSS). Software utilized was Adobe (for pdfs), Microsoft Word, and Microsoft Excel. This line-by-line evaluation identified overarching concepts within the data and provided the initial code book. After finalizing the code book, one investigator (SRW) recoded all data and then presented the results to the qualitative data analysis team (SRW, SSS, HCW, MAG) for interpretation and refinement where we identified patterns and generated initial themes. The entire study team reviewed these themes, named them, and defined them to arrive at the final list of five factors. Arora’s Theoretical Framework of Handoffs, [18–21] generated from handoff experiences within a single specialty night float system, was utilized as a sensitizing concept during the deductive phase, informing our expanded conceptual framework.

**Reflexivity/triangulation/trustworthiness.** We sought to increase reflexivity in our analysis by including multiple investigators with differing perspectives with which to view the data [32, 33]. Three investigators (SSS, JSP, AT) have extensive training and experience in qualitative and quantitative research. Three investigators (SRW, HCW, and MAG) are clinician educators with additional expertise in qualitative methods. Five investigators (SRW, HCW, MAG, AD, LK) have institution-wide leadership experience in graduate medical education. Around handoffs, SRW has held both institutional and national leadership roles in transitions of care task forces and has published on this topic. SRW, AD (Designated Institutional Official), and LK (Associate Dean of GME) were engaged in institutional GME level preparation for the upcoming CLER site visit when the initial survey was done and worked together on a wider interdepartmental task force to enhance transitions of care practices. MAG, HCW, and SRW all have expertise in emergency medicine residency program leadership, utilizing handoffs in their ACGME milestones as well as during clinical practice in the emergency department setting. HCW, SSS, and MAG are all engaged in the design and study of novel assessment tools. In addition, SSS has expertise studying the interdependence that exists within and across clinical teams.

In addition, triangulation was emphasized in multiple capacities: 1) by utilizing both quantitative and qualitative data, 2) by involving multiple investigators in the coding and data review, and 3) by collecting data across multiple specialties and stages of training [34].

To further increase trustworthiness, our survey design was iterative and included member checks for both content and ease of use with an interdisciplinary and interdepartmental group including trainees, nurses, faculty, educators, and administrators both within and outside our institution. We also took great care to design the survey itself to enhance psychological safety as well as to safeguard anonymity to allow trainees to express their thoughts.

**Ethics.** The Stanford School of Medicine Institutional Review Board determined the project did not meet the Federal definition of research. The University of Illinois at Chicago Institutional Review Board determined the study to be exempt.

## Results

**Demographic characteristics.** Our survey response rate was 60.4% (687/1138 eligible respondents). Approximately half of the training programs at our institution were represented in our participant cohort (46/95, 48%), and there were respondents from all post-graduate training years, representing over 30 specialties (Table 1).

**Table 1:** Distribution of Trainee Respondents by Self-Identified Specialty (n = 687)

Training Program	% of Total Responses	Training Program	% of Total Responses	Training Program	% of Total Responses
Anesthesia	54 (7.8%)	Neurosurgery	19 (2.7%)	Radiology	25 (3.6%)
Dermatology	15 (2.2%)	Obstetrics & Gynecology	18 (2.6%)	Neuroradiology	5 (0.7%)
Emergency Medicine	44 (6.4%)	Ophthalmology	3 (0.4%)	Surgery (General)	21 (3.1%)
Medicine	99 (14.4%)	Orthopedic Surgery	18 (2.6%)	Plastic Surgery	9 (1.3%)
Cardiology	9 (1.3%)	Physical Medicine & Rehab.	25 (3.6%)	Thoracic Surgery Integ.	11 (1.6%)
Critical Care Medicine	9 (1.3%)	Otolaryngology	11 (1.6%)	Urology	14 (2.0%)
Gastroenterology	1 (0.2%)	Anatomic & Clinical Pathology	32 (4.7%)	Other Medical	45 (6.6%)
Infectious Diseases	6 (0.9%)	Pediatrics	52 (7.5%)	Other Surgical	10 (1.5%)
Nephrology	9 (1.3%)	Pediatric Cardiology	10 (1.5%)	Other Pediatric	32 (4.7%)
Oncology	8 (1.1%)	Psychiatry	27 (4.0%)	Other Program (optional)	49 (7.1%)
Pulmonary/Critical Care	10 (1.5%)	Child Psychiatry	9 (1.3%)	Declined to state	137 (20.0%)
Neurology	19 (2.7%)	Radiation Oncology	10 (1.5%)		

**Selected response questions.** Most respondents replied “consistently performed” to survey items about handoffs that highlighted the sickest patients, were given verbally and concisely, demonstrated active listening skills by respondents, and were learned informally from senior physicians. Other content was less consistently included. Only two-thirds of respondents reported “consistently performed” about handoffs that mentioned a patient’s code status if NOT full code, easily distinguished between patient care issues, and had checks for confirmation of understanding. Over half consistently highlighted action items and included contact phone numbers.

**Fig. 1:**
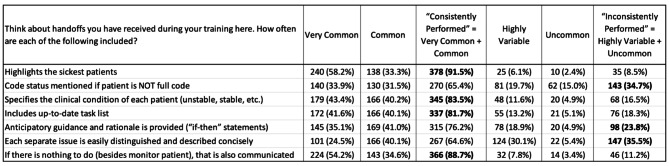
Handoff Communication Content and Frequency of Inclusion (Answered: n = 413)

Findings around duty hours, feedback, and handoff content were revealing. Duty hours and the complexity of patient cases did not interfere with handoffs for most respondents. Respondents generally received informal feedback about their handoffs. Figure 1 describes content that was included in handoff communications, and the frequency of how often and how consistently that content was included. Bolded entries identify elements that were consistently included > 80% of the time, or inconsistently included > 20% of the time, to serve as a priority indicator.

Around handoff systems and structure, most respondents rated as “inconsistently performed” to the use of standardized handoff tools and mnemonics, documentation of handoffs in the electronic medical record, verbal handoffs at the bedside or with family present, attending physician or other healthcare staff participation in handoffs, and the receipt of formal feedback about their handoffs. Standardized handoff tools were acceptable to just over half of respondents, though many qualified that the tools would need to be tailored to their specialty workflow for them to be widely adopted. Handoffs were complicated by patient care demands, lack of private space, lack of available computers, and lack of available staff. Respondents generally received their handoff education informally or through occasional housestaff conferences, and they received little formal feedback about their handoff skills. Half of the respondents did not want to change current handoff practices. Table 5 includes recommendations for institutions based on our findings.

**Fig. 2:**
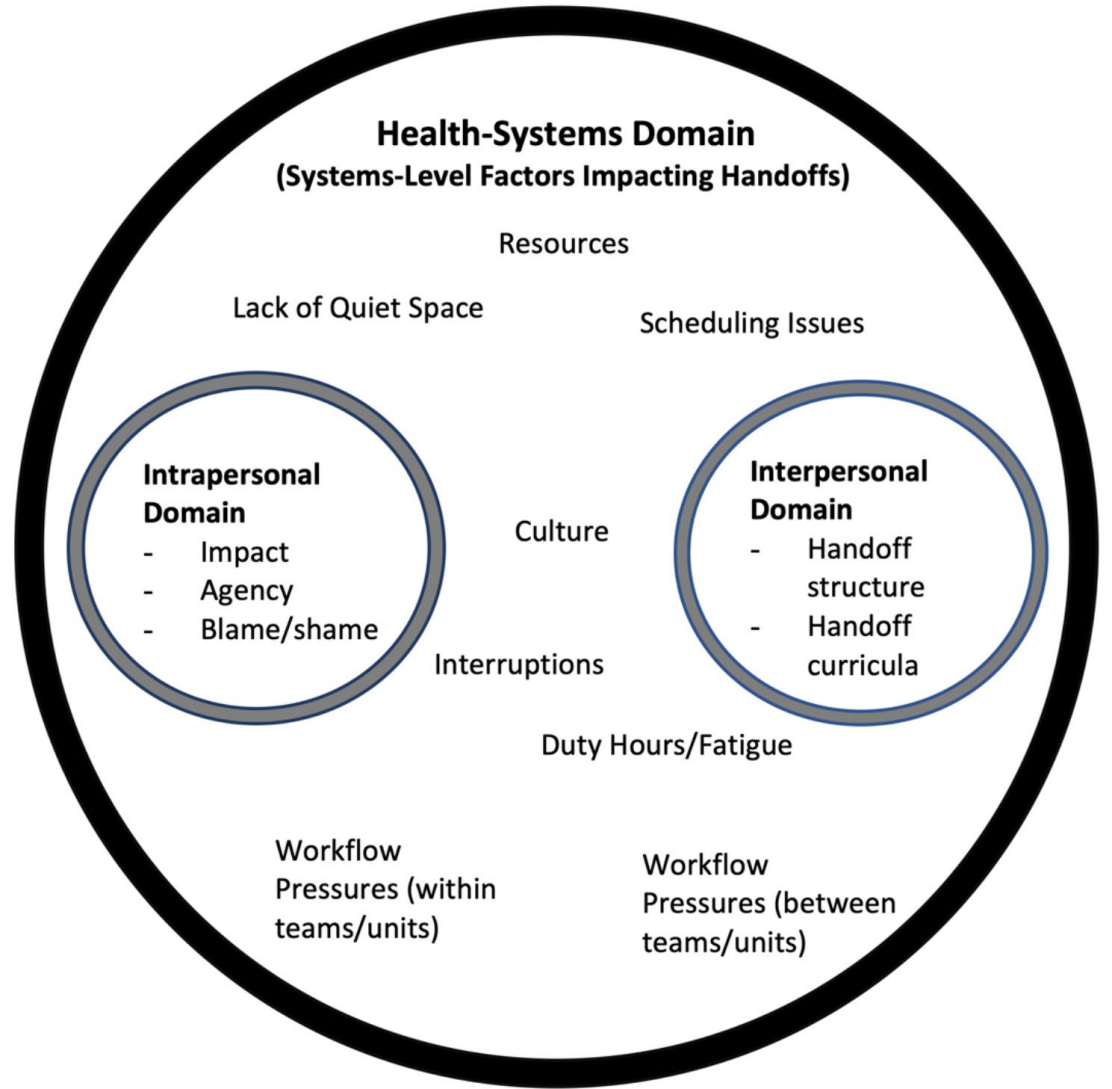
Expanded Theoretical Framework of Effective Patient Handoffs: Intrapersonal, Interpersonal, and Health-Systems Domains

**Qualitative Analysis.** We found wide variability in handoff content and process, revealing issues with both the stakeholders and the health systems in which they function. Our thematic analysis of open-ended responses revealed 5 themes: 1) handoff elements (including factors related to learning handoff elements); 2) health systems-level factors, 3) impact of the handoff, 4) agency (duty), and 5) blame and shame. We define these themes below and provide representative quotes in Table 3. In addition, we suggest an expanded conceptual framework around handoffs informed by our findings (Fig. 2).

**Table 2:** Representative Quotes of Trainee Handoff Experiences for Theme 1: Handoff Elements

*Theme 1: Handoff elements (including factors related to learning handoff elements)* *Definition: how both the content and construct of the handoff communication determine its effectiveness*	
*a) Handoff Structure: Content Suggestions (Please also see* Fig. 1*)*	
“Code status was left out. This led to confusion when the patient became unstable overnight” “Things NOT to change overnight” “Being comfortable with hand-off being a conversation is important, I think” “Who to call if there is a problem with the patient” “Family phone numbers so we don’t have to look for them in Epic… the contact listed…is often not the best one”	
*b) Culture: Role Modeling, Stressing Importance of Handoffs*	
“Culture of senior residents is most important” “Continued reinforcement about proper hand-offs from the Attendings” “They’re important! interns need help with them!” “The best way to learn good handoff/signout technique is to have a list of all pertinent information that must be given during the signout… It should be taught at the beginning of each year and then refreshed at least once during the year. Many people dismiss this type of teaching because they say it’s “intuitive” or “obvious,“ however I’ve noticed that not teaching this leads to messy and unorganized handoffs”	
*c) Formal Training: Training Imbedded in Formal Curricula*	
“I-PASS study training” (multiple mentions) “Handoff lecture, principles of dealing w/ common field-specific problems”	
*d) Observation: Learning Through Observing Others or By Being Observed*	
“Having the chief resident on a service demonstrate the type of handoff they expect on the first or second day of the rotation (although this is difficult since we all start each rotation on different days)” “I used to have my interns check each other’s sign-outs (especially later in the year) and ask each other questions about things that weren’t clear, or issues that they anticipated arising overnight. It helped them improve their own sign-outs for that day, and practice giving/receiving a helpful sign-out”	
*e) Experiential: Learning Through Trial and Error*	
“Nothing can compare to living the experience of good and bad sign out. You can’t teach it because you have to actually live out the effects of things going poorly to know how to improve… you can’t know what you don’t know”	
*f) Lack of Training: Perceived Lack of Training or Wish List Items for Curricula*	
“None. Figured it out on my own during medical student clerkships and intern year” “The lack of a structured sign out formula so people from different services working together don’t speak the same language” “Having a handbook for trainees that contains a written policy and is frequently updated. This gets around the issue of multiple people doing things multiple ways, each claiming that their way is correct”	
*g) Feedback: Examples*	
“Coaches provide us feedback”; “Formal intermittent observation” “Run the signout by the senior resident daily to get feedback on making it better (this improves patient care first and foremost)” “Feedback should be done between residents… I like to ask, ‘anything I missed and should have told you?’ the next morning when I cover my service again… Assessing sign outs more formally is very difficult as these… happen 365 days of the year, between a huge variety of residents in a huge variety of settings. Active feedback between residents is the most important way to help them improve and the best way to actually get honest and valuable feedback”	

**Table 3:** Representative Quotes of Trainee Handoff Experiences Informing Theme 2: Health Systems Level Factors

Theme 2: Health systems-level factors impacting handoffs Definition: trainees describe a variety of systems issues impacting handoffs	
a) Handoff Policies and Protocols: Suggestions	
“Biggest issue for me is that I forget to mention things. With a sign out template, this would be diminished” “A standardized format of what needs to be communicated in list format would ease the flow and make signouters less random” “Have an order in Epic that the accepting team must submit by a certain time that indicates they have accepted care of the pt… nurses will understand the transfer of orders and care has been made”	
b) Handoff Workflow Pressures: Within Teams/Units	
“Too many patients to sign out to one provider” “Too many patients to go into any real detail” “Feel strongly that our model of resident-to-resident handoff with chief resident supervision when possible is an effective and efficient model” “… in high-stakes environments like the ICUs or ED, a fellow or attending should be present for sign out” “People are often spread out through the hospital and hard to reach”	
c) Handoff Workflow Pressures: Between Teams/Units	
“Disconnect between nursing timing, MD timing and bed control timing” “…transfers are often a complete mess, especially when they happen in the middle of the night. The nurses page the wrong team probably about 25% of the time for new admits/transfers” “I think instituting a two-check system whereby nursing/nursing passoff and MD/MD passoff has occurred before transport is called to transition a patient to another level of care is appropriate and indicated for all in hospital transfers…”	
d) Scheduling Issues Impacting Handoffs	
“There should be protected blocks of time which the receiving team should not be paged about transfers because it interferes with rounding or sign out” “Someone to cover the pager during signout”	
e) Environmental Pressures: Interruptions/Lack of Quiet Space	
“No quiet, private place to sign out, so we end up signing out in noisy rooms with lots of distractions” “Workrooms are not large enough”	
f) Environmental Pressures: Duty Hours/Fatigue	
“I don’t know if other people realize how important it is. They just are tired at the end of the day and want to go home (not so much duty hour limits)” “We will go over duty hours to signout appropriately…” “… sometimes switching service means you need to handoff and receive signout on the same day… each process will take 2–3 hours, adding on hours and hours of work, and this does not count towards ACGME hours because “you are not in the hospital”	
g) Additional Needed Resources	
“Need more computers…” “Hospital-sponsored call center to field and triage outpatient calls…” “Electronic system for handoffs, where they can be updated easily and frequently”	

**Theme 1: Handoff Elements**. Our trainees described how both the content and construct of the handoff communication determined its effectiveness. The best handoffs transferred enough information to develop a shared mental model of the patient. Examples of critical handoff content included a case summary, an appraisal of illness severity, the patient’s code status, and tasks to be completed. Receivers were expected to synthesize the content provided, with ample opportunities to confirm understanding and ask questions. Trainees also focused on the importance of situational awareness as a construct, noting that effective handoffs provided anticipatory guidance, if-then action statements, escalation plans, and contact information. Representative quotes are provided in Table 2. 

**Theme 2: Health-Systems Level Factors.** Respondents anchored on numerous systems issues that impacted their handoffs. They identified impediments to effective handoffs that were common in the clinical learning environment, which included high patient volume and acuity, frequent interruptions, and a lack of a quiet space for handoffs. Importantly, they noted that the workplace culture itself could easily exacerbate these barriers. Trainees desired certain resources to facilitate better handoffs including formal training, protected time from other duties, participation by key personnel, and standardized workflows and policies. Issues related to duty hours and fatigue were also described. Representative quotes are provided in Table 3.

**Theme 3: Impact.** The respondents described the impact of effective handoffs in two ways. First, good handoffs resulted in better patient care; therefore, they were inherently important. But some respondents also shared their emotional reactions to handoffs, good and bad, demonstrating vulnerability in their responses. Handoffs impacted their self-notion and self-worth as a physician, and they evoked a clear sense of responsibility in our trainees for the patients under their care. Representative quotes are provided in Table 4, Theme 3.

**Table 4:** Representative Quotes of Trainee Handoff Experiences Informing Themes 3, 4, 5: Impact, Agency/Duty, Blame/Shame

*Theme 3: Impact of the handoff* *Definition: trainees describe the impact of handoffs on them, both in how handoffs support their role as physicians as well as the emotional impact of both good and bad handoffs on them*	
*Positive Impact*: “A resident went through every patient on the list and specifically mentioned what the most likely things to go wrong were and what to do in those scenarios. The most prepared that I’ve ever been” *Negative Impact*: “Too many patients, it was overwhelming. I felt as though I was in an outer worldly experience. My first ever time receiving sign out” “… I have assumed care of a newly intubated patient with absolutely no changeover from the fellow who responded to the code… I needed to start all lines and put in orders immediately. I had…no help, it was 1 h before rounds … I had never initiated {x therapy}… before, and that was the most traumatic experience of my residency thus far” “Experiencing panic due to bad signout”	
***Theme 4: Duty (Agency)*** *Definition: professionalism concepts associated with patient handoffs, most importantly around respondents acting in the best interests of their patients*	
*Positive Valence*: “There is always time to give a good sign out. Residents need to prioritize this as part of our job responsibility” “When the …resident signed out to me … I didn’t understand this sign out … I decided to go through the chart myself. I was worried about the patient and did not move them out of {unit}… later…the patient coded. It reinforced that if a sign out doesn’t make sense, you should look through the chart more carefully yourself” “Previous doctor was honest upon uncertainties about the patient which allowed me to take a fresh approach… rather than having a closed mind in regards to diagnosis” “…I appreciate that my attending was always available and even though we did have a bad outcome with patient, we were on same page about management… and… goals” *Negative Valence*: “When on call covering numerous patients you don’t know well, there will invariably be information of situational importance not included in the signout. There is simply no substitute for “knowing” the patient” “There’s nothing to sign out. Call if?s.“ I called & said that wasn’t ok. The person frustratedly ran through each pt on list w me…and if/then plans were discussed that had not been written on signout”	
***Theme 5: Blame and shame*** *Definition: blame is when providers are assigned responsibility for an error due to a perceived fault or wrong related to a handoff. Shame is described as painful feelings of humiliation or distress related to poor handoff experiences*	
“Laziness and arrogance of the residents” “Some colleagues who don’t put effort into it” “I remember receiving a handoff… where the resident was in a hurry to leave… There wasn’t any time to figure out the patient’s PMH, fluid balance, or … course– so while I was able to proceed…and the patient did fine, I was far outside my comfort zone. It could have been harmful to the patient, and certainly damaged my relationship with that resident, whom I didn’t trust again” “At sign-out we were briefly told to treat if… without specific instructions…We managed the patient given the nebulous information we had. To our dismay, the morning after, …{colleagues} were disappointed with our management strategies overnight. This affects our therapeutic alliance with consultants and with the patient” “Patient was signed out as stable. In reality, this person had … [condition] ongoing for hours that was not well controlled nor well signed out. I called the resident to ask for clarification and this person came back to the hospital. I did not request this specifically but I think this person felt bad about it. Patient care was not really affected given the easy communication between residents”	

**Theme 4: Duty (Agency).** Several professionalism concepts were associated with patient handoffs, most importantly that the respondents acted in the best interests of their patients. Some respondents described a professional duty to ensure effective handoffs, akin to the concept of agency in Arora’s framework [18, 20]. They reported accountability to the patient and having a sense of ownership of the patients’ care. Respondents also attributed these professional duties to teams of providers engaged in handoffs, citing the need for teamwork and a team mentality during handoffs. Representative quotes are provided in Table 4, Theme 4.

**Theme 5: Blame and Shame.** Trainees recounted instances of blame, when providers assigned responsibility for an error to a perceived fault or wrong related to a handoff. These faults extended beyond content errors; they included judgments about provider behaviors and character, as well as unmet or unequal expectations. Quotes that are particularly relevant here are provided in Table 4, Theme 5. This example describes the lasting impact on the relationship between trainees: “I remember receiving a handoff… where the resident was in a hurry to leave… There wasn’t any time to figure out the patient’s PMH, fluid balance, or … course– so while I was able to proceed…and the patient did fine, I was far outside my comfort zone. It could have been harmful to the patient, and certainly damaged my relationship with that resident, whom I didn’t trust again.”

Conversely, shame was also evident in the responses, as respondents described painful feelings of humiliation or distress related to poor handoff experiences. Examples included guilt around perceived mistakes related to a handoff and negative self-assessments of competence. An example: “At sign-out we were briefly told to treat if… without specific instructions…We managed the patient given the nebulous information we had. To our dismay, the morning after, {colleagues} were disappointed with our management strategies overnight. This affects our therapeutic alliance with consultants and with the patient.”

These five themes were inductively identified from our thematic analysis. Our expanded conceptual framework deductively utilized the lens of Arora et al.’s framework [18] and incorporated the additional themes we identified. Coordination costs and agency theory are still certainly relevant. For example, agency/duty shows up strongly as one of our themes (in the intrapersonal domain). Coordination costs are highlighted in both handoff structures (interpersonal) and workflow pressures (health-system). Our trainees’ perspectives and experiences highlighted that safe and effective handoffs are only partially within their control, though they impact trainees greatly.

## Discussion

Our findings inform an expanded theoretical framework of effective patient handoffs, which includes three domains: health systems, interpersonal, and intrapersonal (Fig. 2). This builds on the important work of Arora et al. [18].Solutions to the continued patient safety threats that stem from suboptimal handoffs need to address all three domains to be effective and sustainable.

**Expanded Framework for Patient Handoffs.** We view these findings as representing three domains that provide a wider and more comprehensive exploration and framework for patient handoffs: 1) an Intrapersonal Domain: comprised of factors that the individual controls, which include professionalism, engagement, preparation, emotions, and agency, among others; 2) an Interpersonal Domain that includes all interactions and communications between providers during a patient handoff, as well as the educational curriculum that informs it; and 3) a Health-Systems Domain that consists of systems-level factors, cultural influences, and the complexities of the clinical learning environment within which both other domains must operate (Fig. 2).

Studying handoff experiences across an entire institution allowed us to identify health systems-level factors and cultural issues that can impact safe handoffs and, yet, are often underexplored. Our framework highlights the importance of factors that are not easily addressed by individual providers and would benefit from interventions by both training programs and sponsoring institutions. It also emphasizes the danger of assessing trainees without accounting for the permeating health-systems factors that may negatively impact their successes [35–37]. Deliberate attention, support, and resources are necessary to address this phenomenon. Otherwise, improvements are unlikely, and it will be difficult to disentangle the impact of faulty systems on trainee performance and patient safety outcomes.

Our respondents provided a rich inventory of recommendations and strategies for improving patient handoffs. This inventory should be viewed by educators and administrators with a sense of urgency because it represents an inventory of trainee needs – needs for new protocols, education, and infrastructure to help them be successful in their clinical duties. These recommendations are actionable and summarized in Table 5. Though similar lists of recommendations and handoff protocols are available in the literature, very few have been generated from the perspectives of trainees and none have represented such a wide range of specialties [38–43].

Systems-level recommendations include providing the means for reducing distractions, improving resources (such as space and computers), and protecting housestaff time. As for oversight, respondents recommended that a fellow or attending be present during higher stakes handoffs such as those in the intensive care unit or emergency department. Our trainees identified the importance of teams; for our construct, pressures within and between teams and units are housed within the health systems domain. Many other factors are of course at play, including issues of autonomy and local departmental culture. These findings compliment the teamwork model for handoffs developed by Webster et al., which considers theories related to organizational psychology, systems engineering, and human factors [44].

Additional health-systems level protections were suggested for transitioning patients from one team to another. Respondents identified poor or no handoffs between teams, misaligned nurse-physician-bed control workflows, and nighttime transfers as opportunities for health systems to improve structured handoffs.

Other suggestions included enhanced functionality of electronic medical records to make it easier to update handoffs and to clarify the care teams responsible after a handoff. Most respondents supported the adoption of a hospital-wide, standardized patient hand-off tool or protocol, as long as it could be customized to individual services. However, there was resistance from participants to having a tool “forced on us,” emphasizing the importance of culture and stakeholder buy-in.

**Table 5:** Trainee-Informed Recommendations for Health Systems to Improve and Support Handoffs

*Health-Systems Domain*	
Providing physical environment that supports safe handoffs	
Protecting sign-out area and personnel from interruptions/distractions Blocking time specifically for handoffs Providing adequate workspaces, including sufficient computers with electronic medical record (EMR) access	
Personnel	
Right-sizing teams participating in handoffs Including appropriate supervision in higher stakes handoffs (to include fellow or attending)	
Between-team and between-unit transitions	
Ensure handoffs occur when a patient is transferred from one team/department to another Align nurse-physician-bed control workflows Clarify additional operational supports for night-time transfers	
Electronic medical records	
Design enhanced functionality of electronic medical records to make it easier to update handoffs Clarify the specific care teams responsible after a handoff Adopt standardized patient hand-off tool that can be easily customized to individual services	
*Interpersonal Domain (handoff structure/handoff curricula)*	
Provide all patient care providers with key content to include in patient handoffs Provide institution-wide training in hand-off best practices Provide specialty-specific training in hand-off best practices Enhance feedback mechanisms around handoffs (including peer-peer feedback)	
*Intrapersonal Domain (impact, agency, blame/shame)*	
Acknowledge stressors related to handoffs Acknowledge the complex system in which handoffs occur and partner with trainees on improving it Encourage well-being interventions that mitigate blame/shame mindset	

As regards the interpersonal domain around the handoff communication itself, we found wide variability in the implementation of handoff curricula, ranging from none in many programs to SBAR [25, 26] and I-PASS [12, 27] training in others. Learning how to do handoffs from senior residents, fellows, or faculty while on clinical service was common; our findings suggest that these interactions likely aculturalized the respondents to the importance of handoffs. Yet, it is clear that institution-wide training is needed to supplement the clinical teaching of handoffs, as our respondents reported that best practices were inconsistently performed. Numerous handoff curricula are described in the literature for this purpose. [26, 27, 39, 45]. It is also clear that programmatic interventions are needed to improve feedback to housestaff about their handoffs. Feedback from peers and near-peers should be strongly considered, especially given the reported absence of faculty members during handoffs. Feedback rubrics are available in the literature [46].

Lastly, as it relates to the intrapersonal domain, our respondents largely described how handoff duties evoked deep senses of duty and responsibility to patients, in contrast to the ‘agency problems’ predicted by Arora [18]. Specifically, respondents described remaining past their duty hours limits in order to complete handoffs. Our respondents repeatedly acknowledged the importance of patient handoffs in many different ways, and they made clear their desire to do them properly.

While positive handoff experiences were noted by many respondents, the descriptions of negative handoff experiences and their impacts and emotional reactions are concerning. Representative quotes from our impact and blame/shame themes include: “That was the most traumatic experience of my residency thus far”, “Experiencing panic due to bad sign-out”, “Damaged my relationship with that resident, whom I didn’t trust again”, and “To our dismay, the morning after, …{colleagues} were disappointed with our management strategies overnight.” Avoidance of negative emotions during residency may be a motivator for trainees to learn and perform [47, 48]. However, when experienced, these emotions threaten to negatively impact physician well-being and feed into burnout [49]. Our study adds negative handoff experiences to an emerging literature on constructs and systems within medicine that have blame and shame undercurrents, such as morbidity and mortality conferences and inappropriate use of the Socratic method (“pimping”) [50, 51]. Bynum and team describe shame experiences as sentinel emotional events during training [50]. These issues must be addressed if we are to be successful at fostering a true growth mindset in medical education [52].

The degree of autonomy our trainees experienced in managing handoffs was highly variable, with some expressing a sense of fear and dread in anticipation of the task. Trainees’ self-perceptions of their competence as physicians are often impacted by factors that are largely outside of their direct control, such as the health-systems issues that we identified [53, 54]. Holden discusses the danger of attributing blame and causality for poor handoffs to individuals rather than to health systems [53]. Housestaff also lack the experience necessary to develop comfort with medical uncertainty and are just starting to build illness scripts that reflect expected disease progression; these may impact the confidence and efficacy of junior trainees. The perfection they expect of both themselves and their colleagues with respect to handoffs is unrealistic and damaging given the realities of our health systems. Institutional interventions are needed to protect trainee well-being, and educators must facilitate a cultural shift away from the blame and shame that permeates the clinical work environment [49–54].

There are some limitations to this study. All survey studies are inherently subject to sampling errors and biases that may affect the validity and generalizability of the results. However, we believe our institution-wide sample was robust given the response rate and participant representation of many specialties across all training years. Phillips, Reddy, and Durning note that a response rate of > 60% has been described as suggesting a lower probability of nonresponse bias [30]. That being said, it remains important to consider the implications of potential nonresponse bias in our findings. One example would be subgroups of trainees who may not perform many handoffs in their work. If included, their perceptions might appear to dilute the experiences of those for whom handoffs are a central element to their lived experience.

Participant responses may have been subject to recall bias, as the survey asked about personal experiences which may have occurred earlier. This may have skewed our results toward more negative responses. Several other common response biases may be amplified in studies with trainees; these include acquiescence bias, social desirability bias, and extreme response bias. It is difficult to predict how such biases may have impacted our data. Nevertheless, our data set represents an important and robust perspective from trainees from over 30 specialties at a large academic medical center.

LaDonna, Taylor, and Lingard note that for “data to be “rich,” they must have context, personal meaning, emotional and social nuances, and layers of detail” [55, p. 347]. Despite being an open-ended survey, the richness of our narrative feedback certainly reflects each of these components. In addition, providing this opportunity for expression in a survey form allowed us to gain perspectives from a large, multispecialty population of trainees who are incredibly busy and may not have the bandwidth to opt into focus groups or other more time-intensive qualitative methodologies. Our approach supported inclusion through hearing the stories of a wide range of trainees and allowed us to get a glimpse into commonalities and differences of their shared experiences. This also provided great context to the quantitative responses; both complemented the overall analysis. Our findings open the door to future qualitative studies, informing next steps and even deeper dives.

## Conclusion

In summary, our findings indicate that health systems, interpersonal, and intrapersonal issues affect handoff communication. We recommend an expanded theoretical framework for effective patient handoffs and provide a set of trainee-informed strategies that training programs and sponsoring institutions should consider implementing and studying as we continue to explore avenues to protect patient safety and enhance clinical outcomes.

There is a need for institution-wide training of providers that emphasizes the process, monitoring, and outcomes of handoff communications. Cultural and health-systems issues must be prioritized and addressed, as an undercurrent of blame and shame permeates the clinical environment. Further study is warranted once programmatic and institutional interventions occur.

## Electronic supplementary material

Below is the link to the electronic supplementary material.

Additional Files: The complete GME survey is available as Appendix A, GME Housestaff Survey

## Data Availability

The datasets generated and/or analyzed during the current study are not publicly available due to it being an institutional QI study. Relevant quantitative and qualitative data are reported in the manuscript and/or supplemental materials.

## List of abbreviations

ACGME: Accreditation Council for Graduate Medical Education
AMD: Ann Dohn
AT: Ara Tekian
CLER: Clinical Learning Environment Review
ED: Emergency Department
EMR: Electronic Medical Record
Et al.: Et alia (and others)
HCW: Holly Caretta-Weyer
ICU: Intensive Care Unit
Integ.: Integrated
I-PASS: Illness severity, Patient summary, Action list, Situation awareness and contingency planning, and Synthesis by receiver
YSP: Yoon Soo Park
LK: Laurence Katznelson
MAG: Michael Albert Gisondi
MD: Medical Doctor
RN: Registered Nurse
SBAR: Situation, Background, Assessment, and Recommendation
SRW: Sarah Roberts Williams
SSS: Stefanie Sebok-Syer
QI: Quality Improvement
