# Supplementary material for: Patient handoffs and multi-specialty trainee perspectives across an institution: informing recommendations for health systems and an expanded conceptual framework for handoffs
Source: BMC Med Educ. 2023 Jun 13;23:434. doi: 10.1186/s12909-023-04355-5 (PMC10262514; doi:10.1186/s12909-023-04355-5)
Supplement: Supplementary file 1 — Additional Files: The complete GME survey is available as Appendix A, GME Housestaff Survey [file 12909_2023_4355_MOESM1_ESM.pdf]

Better Handoffs, Safer Patients

Improving patient handoffs and sign-outs, also known as "transitions of care", has been shown to significantly enhance patient safety. We are surveying you, the housestaff, because you are a critical component of the day-to-day care of our patients. Your insights are vital.

We want to understand how handoffs take place in your department and between units. We also hope to identify barriers to optimal handoffs. We plan to use the results to obtain resources that will enhance the safety of our patients and improve your experience.

The Transitions of Care Task Force represents multiple specialties (including medicine, surgery, pediatrics, anesthesia, neurosurgery, and emergency medicine). It includes residents, faculty, nursing, and hospital leadership. A huge thanks to them for their help designing this survey and to you for taking it.

Sincerely,

Laurence Katznelson, MD, Professor of Neurosurgery & Medicine, Associate Dean of GME
Ann Dohn, MA, Director, GME, Stanford D.I.O.
Sarah R. Williams, MD, Emergency Medicine, Chair, Transition of Care Task Force

Survey Instructions and Informed Consent

Results of this survey will not affect your academic or professional standing. Responses will be anonymous and confidential. Information will be aggregated to protect anonymity.

Based on pilot testing this should take about 15 minutes to complete. You can exit and go back to it at any time until you hit "Done".

Text boxes are often provided. Their use is encouraged but optional depending on your time constraints.

◯ Informed Consent: By clicking the radio button to the left, you affirm that you have reviewed the information on this and the previous page and that you agree to participate in this survey. This survey has been reviewed and cleared by the IRB. Thank you for your time.

Were you a resident or fellow during the academic year just ending (2013-14)?

◯ Resident ◯ Fellow

What year of training were you in during academic year 2013-14?

- ◯ Medical School
- ◯ PGY 1
- ◯ PGY 2
- ◯ PGY 3
- ◯ PGY 4-6
- ◯ PGY 7-9

Other (please specify)

During 2013-14, what residency or fellowship were you in? Small programs are not listed in order to protect anonymity. In this case, please select "Other Medical", "Other Surgical", or "Other Pediatric" from the drop down list. If you would like, you may identify it below (optional).

Other program (optional)

Transitions of care (also known as "sign-outs" or "handoffs") are defined as “a real-time process of passing patient-specific information from one caregiver to another or from one team of caregivers to another for the purpose of ensuring the continuity and safety of the patient’s care." (1)

THIS SURVEY FOCUSES ON PATIENT HANDOFFS BETWEEN HOUSESTAFF (including residents and fellows).

These commonly occur:

- at the end of a work period or shift
- when a patient is transferred from one team to another
- at the interface between a consult team and the primary care team

Think about a memorable experience you had when you received a patient handoff. Please describe what went well or poorly. How did it affect patient care? How did it affect you? (No names please.)

How did you learn about safe and effective patient sign-outs? Check all that apply.

- ☐ From more senior residents, fellows, and faculty while on clinical service
- ☐ During a handoff lecture during intern year
- ☐ A series of lectures throughout training
- ☐ Discussed during M&M, QA/QI, etc.

Suggest other good ways of learning this?

The following are potential barriers to safe and effective handoffs. How often has each impacted you?

	Very common	Common	Highly variable	Uncommon
Duty hour limits (need to hurry or risk being in violation)	○	○	○	○
Workload: too many patients (not enough time to give good sign-out for each)	○	○	○	○
Acuity: patients very sick and/or complicated (comprehensive sign-outs are difficult)	○	○	○	○

Other barriers that have impacted you?

Think about handoffs you have received during your training here. How often are each of the following included?

	Very common	Common	Highly variable	Uncommon
Highlights the sickest patients	○	○	○	○
Code status mentioned if patient is NOT full code	○	○	○	○
Specifies the clinical condition of each patient (unstable, stable, etc.)	○	○	○	○
Includes up-to-date task list	○	○	○	○
Anticipatory guidance and rationale is provided (“if-then” statements pending results of tests/ interventions /reassessments)	○	○	○	○
Each separate issue is easily distinguished and described concisely	○	○	○	○
If there is nothing to do (besides monitor patient), that is also communicated	○	○	○	○

Think about when you receive VERBAL sign-out. How often are the following done?

	Very common	Common	Highly variable	Uncommon
The physician signing out to me uses concise, concrete language	◯	◯	◯	◯
They check in with me that I understand the plan	◯	◯	◯	◯
If verbal and written sign-outs are both given, they are consistent with each other	◯	◯	◯	◯

How often do the following apply to WRITTEN sign-outs you receive?

	Very common	Common	Highly variable	Uncommon
The note has an active problem list including 1) abnormal vitals, 2) overall patient stability, 3) known diagnoses, 4) concerning symptoms if diagnoses unclear, and 5) abnormal lab or procedure results	◯	◯	◯	◯
Important contact phone numbers are included and kept up-to-date in the note or EPIC	◯	◯	◯	◯

Now think about when you hand off your patients to others. How often do the receivers of your sign-out do the following?

	Very common	Common	Highly variable	Uncommon
They confirm understanding of my sign-out	◯	◯	◯	◯
They appear focused, engaged, and demonstrate active listening skills	◯	◯	◯	◯
They verbalize a concise, accurate summary of each patient	◯	◯	◯	◯

What other important content should be included in patient sign-outs (verbal or written)?

How do you get feedback about your patient handoffs? Please check all that apply.

- [ ] I don't get specific feedback about handoffs
- [ ] Informal feedback from colleagues, faculty
- [ ] Feedback from patient handoff evaluation tool(s)
- [ ] Milestones: handoffs are part of our specialty Milestones
- [ ] My program director and/or others in the program leadership give me feedback

Other suggested useful feedback methods?

When patients are transferred from one team to another (such as ED to inpatient, or ICU to floor), how common are the following?

	Very common	Common	Highly variable	Uncommon
The receiving team clearly states when they will assume care of the patient	○	○	○	○
An active problem list (either written or verbal) is provided by the transferring team	○	○	○	○
The RNs contact the appropriate MDs	○	○	○	○
It is clear which team will be communicating incidental abnormal findings to the patient's primary care physician	○	○	○	○

Suggestions to improve handoffs between units?

Regarding consult services and their coordination of patient care with the primary (including ED) team:

	Very common	Common	Highly variable	Uncommon
Consult recommendations are given verbally	○	○	○	○
Consult recommendations are written in the medical record	○	○	○	○
It is clear who will be writing orders on the patients: the consulting team or the primary team	○	○	○	○
Effective standardized protocols are in place to assist when contradictory recommendations are given by different services	○	○	○	○

Suggestions to improve consultations?

Regarding physician-nurse communication, how do you feel about these issues?

	Agree strongly	Agree	Disagree	Disagree strongly
It is easy to identify and contact the primary or covering nurse taking care of my patient(s) during sign-out	○	○	○	○
It would be helpful for sign-out if nurses batched non-emergent phone calls to particular times of day	○	○	○	○
I would involve nurses in handoffs more often, but it is logistically difficult (please describe why below)	○	○	○	○

Regarding communication with nurses:

What has worked well for you? __________

What has been frustrating? __________

How has the electronic medical record affected nurse-physician communication? __________

Do conversations happen with the patients and/or families during handoffs?

- ☐ Yes, we routinely involve them in handoffs
- ☐ Yes, sometimes
- ☐ No, not usually

Regarding involving patients in handoffs:

If you involve patients, what works well? __________

If you don't, what gets in the way? __________

Besides the resident or fellow you are signing out to, how often are the following personnel present during your handoffs?

	Very common	Common	Highly variable	Uncommon
Attending	◯	◯	◯	◯
Supervisory resident or fellow	◯	◯	◯	◯
Charge nurse or patient's nurse	◯	◯	◯	◯
Case managers/social workers, etc.	◯	◯	◯	◯

Any suggestions about team composition during handoffs?

How often do the following circumstances impact handoffs?

	Very common	Common	Highly variable	Uncommon
Lack of a protected space free of distractions	◯	◯	◯	◯
Lack of sufficient computer terminals	◯	◯	◯	◯
Availability of team members	◯	◯	◯	◯

What are additional needed resources/personnel?

How often do you use the following techniques to sign-out?

	Very common	Common	Highly variable	Uncommon	N/A
Verbal: face-to-face in a workroom/space	◯	◯	◯	◯	◯
Verbal: during bedside rounds	◯	◯	◯	◯	◯
Verbal: by phone	◯	◯	◯	◯	◯
Written sign-out note in EPIC	◯	◯	◯	◯	◯
Written sign-out note (not in EPIC)	◯	◯	◯	◯	◯

Any techniques you particularly like?

If your department uses a tool or mnemonic to help standardize the handoff process, please check it below.

- [ ] "SBAR” or "SBARQ"
- [ ] “SIDECHAT”
- [ ] “I-PASS”
- [ ] "SAIF-IR"
- [ ] Other (please specify below)
- [ ] My department has one, but I don't remember which one

Other (please specify)

If your department has a tool or mnemonic, how often do you personally use it during handoffs?

Very common	Common	Highly variable	Uncommon
○	○	○	○

If you do not use it commonly, why not?

Should we adopt a standardized patient hand-off tool or mnemonic to be used throughout the hospital?

- [ ] Yes, a standardized tool would increase patient safety
- [ ] Yes, as long as it can be customized slightly for our particular service
- [ ] No, I like what we have now
- [ ] No, other reason

If "No, other reason", why not?

During your rotations, did you notice any particular departments or units that were particularly good at sign-out? If so, please identify here and briefly explain.

Please reflect on the unit you worked in where the handoffs were the most problematic. Which was it? What would have helped to improve the situation? (Personnel, work-flow, and/or resources)

Any other thoughts or suggestions about handoffs that you haven't mentioned?

Any recommendations on how to improve this survey? Please also feel free to email me at srwilliams@stanford.edu. Thanks very much!

References:

(1) Joint Commission, 2007
